# Analytic Continuation, Phase Unwrapping, and Retrieval of the Refractive Index of Metamaterials from S-Parameters

**DOI:** 10.3390/s24030912

**Published:** 2024-01-30

**Authors:** Giovanni Angiulli, Mario Versaci, Salvatore Calcagno, Paolo Di Barba

**Affiliations:** 1 Department of Information Engineering, Infrastructures and Sustainable Energy, Mediterranea University, 89124 Reggio Calabria, Italy; giovanni.angiulli@unirc.it; 2 Department of Civil, Energetic, Environmental and Material Engineering, Mediterranea University, 89124 Reggio Calabria, Italy; calcagno@unirc.it; 3 Dipartimento di Ingegneria Industriale e dell’Informazione, University of Pavia, 27100 Pavia, Italy; paolo.dibarba@unipv.it

**Keywords:** phase unwrapping, scattering parameters, metamaterials

## Abstract

The heuristic homogenization approach is intensively employed to characterize electromagnetic metamaterials (MMs). The effective parameters are extracted within this framework using the Nicolson–Ross–Weir (NRW) method. Special attention must be devoted to handling this procedure because of the branch ambiguity issue affecting it, i.e., the lack of uniqueness in the evaluation of the effective refractive index $n_{eff}$ rooted in the use of the multivalued complex logarithm to invert the Airy–Fresnel relation. Over the years, several techniques based on the phase-unwrapping approach have been introduced, but without any theoretical justification. In this paper, we aim to clarify the theoretical connection between the phase unwrapping method and the analytic continuation theory framework. Furthermore, three-phase-unwrapping approaches, which descend directly from the theory we discussed, are compared to identify which approach is best suited to reconstruct the complex refractive index of metamaterials when the NRW method is applicable.

## 1. Introduction

Retrieving the electromagnetic parameters of materials is a task of primary importance in many research fields, such as microwave engineering, electromagnetic compatibility, and bioelectromagnetics, to name a few [1,2,3]. In the last decade, researchers have shown great interest in developing particular three-dimensional artificial materials, usually made up of a lattice of metallic resonant inclusions arranged in a dielectric host medium called metamaterials (MMs), given obtaining devices endowed with exceptional operating performances [4]. To characterize MMs, the so-called heuristic homogenization approach, developed for the first time in [5,6], is the procedure most commonly employed by practitioners and researchers in the field [7,8,9,10,11,12,13,14]. In this peculiar framework, it is assumed that an MM, within its operating frequency range, can be considered analogous to a homogeneous medium, called the effective medium, for which the electromagnetic properties are described by an effective electric permittivity $\epsilon_{eff}$ and magnetic permeability $\mu_{eff}$ [5,6]. Such effective permittivities are extracted by processing the numerical or measured scattering parameters of the MM at hand by using the Nicolson–Ross–Weir (NRW) method [5]. Despite the intrinsic plainness of the NRW method, this recovering procedure often results in nonlocal $\epsilon_{eff}$ and $\mu_{eff}$ [15,16], and this poses severe problems regarding its scope of applicability [15,16]. Nonetheless, the complex refractive index $N_{eff}$ provided by the NRW approach can still be used for recovering the effective parameters for one particular class of MMs, called Bloch lattices [15,16,17]. For these MMs, the effective parameters recovered by using $N_{eff}$ in conjunction with the concept of the Bloch impedance $Z_{B}$ are consistent, obeying the locality constraints [17]. More precisely, for this class of structures, the description in terms of $\epsilon_{eff}$ and $\mu_{eff}$ can be extended far beyond the quasi-static limit, reaching the frequency range in which the MM operates [17]. To evaluate $N_{eff}$ correctly, special attention has to be devoted to its recovering to provide correct results because of the branch ambiguity issue affecting the NRW method, i.e., the lack of uniqueness of Re[Neff], and the effective refractive index $n_{eff}$, which is rooted in the use of the multivalued complex logarithm LOG(·) to invert the Airy–Fresnel relation [18]. In the literature, to face this ambiguity problem, several strategies grounded on the phase-unwrapping approach have been introduced [19,20,21], although without providing any theoretical justification for their use [22]. Only recently, the link between phase unwrapping and the operation of analytic continuation of holomorphic functions has been demonstrated [22]. Following a previous couple of works [22,23], in this study, we aim to better clarify the connection between the phase unwrapping method and the analytic continuation framework and identify which way is best suited to reconstruct the refractive index of metamaterial structures. The paper is organized as follows: Section 2 provides the elements of theory needed to relate analytic continuation and phase unwrapping. In particular, in Section 2.1, we summarize the NRW approach; in Section 2.2, we discuss the link between phase unwrapping and the analytic continuation theory and how the branch issue problem affecting the NRW method can be overcome using the phase-unwrapping approach. In Section 2.3, we connect the Kramers–Kronig integrals to phase unwrapping using the concept of the Riemann surface. In Section 3, the numerical performances of the phase-unwrapping procedures introduced in the above subsections are investigated. To this end, three different samples of a μ-negative MM composite realized via a three-dimensional array of LiTaO_3_ spheres embedded in the free space have been considered. A numerical comparison of the phase unwrapping findings with those provided by the K-K relations and the Maxwell–Garnett theory is performed to rank the methods. Finally, in Section 4, conclusions are drawn.

## 2. Theory

### 2.1. The NRW Approach for Recovering the Complex Refractive Index

The Nicolson–Ross–Weir (NRW) method is a standard technique for recovering the permittivity and permeability of linear, isotropic, and homogeneous electromagnetic media [24] through closed-form relations derived considering the reflection–transmission phenomenon involving a plane wave normally incident on a slab of material with thickness *d*, placed in free space. Due to its inherent conceptual simplicity, the NRW method has also been applied as a homogenization method for metamaterials, as discussed extensively in [5,6]. According to this approach, the complex effective refraction index $N_{eff}$ of an MM can be recovered by solving the following relation (the time-dependence convention $e^{-i\omega t}$ is adopted) [22,24]:

(1) $$\begin{array}{c} e^{iN_{eff}k_{0}d}=\frac{S_{21}}{1-S_{11}R}, \end{array}$$

where $k_{0}$, $S_{11}$, and $S_{21}$ are the the free space wave number, and the S-parameters of the MM under study. The reflection coefficient between the effective medium slab and the free space, R, appearing in relation (1) is as follows:

(2) $$R=\frac{z-1}{z+1}\;with\;z=\pm \sqrt{\frac{{\left(1+S_{11}\right)}^{2}-S_{21}^{2}}{{\left(1-S_{11}\right)}^{2}-S_{21}^{2}}},$$

where z represents the MM effective intrinsic impedance. Using the complex logarithm LOG(·)=log(·)+2pπi to solve (1), in which the term log(·) denotes the principal logarithm [25], and  the integer p∈Z represents the branch index, we obtain the complex refractive index $N_{eff}$ as follows:

(3) Neff(ω)=ik0dlogS211−S11R12+2pπi.

From (3), the expressions of the effective extinction factor $\kappa_{eff}$, the effective refractive index, and $n_{eff}$ read as follows:

(4) κeff=−1k0dRelogS211−S11R

(5) neff=1k0dImlogS211−S11R+2pπ.

### 2.2. Analyticity and Phase Unwrapping

To avoid the lack of unicity of the refractive index $n_{eff}$, when solving (1), it is necessary to take into account some preliminary considerations about the invertibility of the complex exponential function $e^{\left(\cdot \right)}$. More precisely, relation (1) will be invertible, and its inverse will be unique if and only if $e^{\left(\cdot \right)}$ is univalent [22,25]. Unfortunately, this property is related to the term $iN_{eff}k_{0}d$, which is the unknown of the problem at hand. Accordingly, we must treat $e^{\left(\cdot \right)}$ as not univalent, and since the inverse function does not exist in this case, we have to solve (1), computing its right inverse [22]. The following theorem provides us with a method for its evaluation [22]:

**Theorem** **1.** *Let $\gamma :\left[a,b\right]\to \dot{C}$ be a continuous path. There exists a unique analytic logarithm L(·) such that:*

(6) $$e^{L(z)}=z\;\forall z\in \gamma \left(t\right)\;t\in \left[a,b\right],$$

*fulfilling the constraint condition Im(L(γ(a)))=θ0 with $\theta_{0}\in R$ and $e^{i\theta_{0}}=\frac{\gamma (a)}{\left|\gamma (a)\right|}$.*

The detailed proof of the above theorem is given in [22]. In the following, we will summarize the rationale and highlight the most essential points. First, the solution of the equation is calculated by superimposing the path γ(t) with a series of disks, thus realizing the so-called path covering [25]. Each disk is the domain of an appropriate analytic logarithm. In this way, the analytic continuation operation is set up. Second, the analytic logarithm solution of (6) results as the appropriate superposition of the analytic logarithms defined within each disk, for which the phase term is made appropriately continuous as it passes from one disk to another. The operation essentially entails the restoration of the continuity of the imaginary part of L(·) that, as an important consequence of Theorem 1, can be obtained through a phase-unwrapping approach. In the particular case of Equation (1), when $e^{\left(\cdot \right)}$ is right-inverted using the above result, it is fundamental to use a suitable constraint condition to obtain a unique significant solution from a physical point of view. Considering that $\gamma \left(t\right)≜S_{21}/\left(1-S_{11}R\right)$ with t=ω, the constraint Im(L(γ(a)))=θ0 has to be specialized as follows:

(7) ImLS211−S11Ra=ω0=0=0,

which ensures the causality of the refractive index [22]. In fact, this choice ensures that ImLγ(t) is an odd function. In this way, we have that L(·) results as the Fourier transform of a time-domain causal physical quantity [22], and that the principal logarithm log(·) can be used as a first element of the chain of analytic function elements realizing L(·). Having calculated the right inverse L(·), $N_{eff}$ is given by the following:

(8) $$N_{eff}=\frac{1}{ik_{0}d}L\left(\frac{S_{21}}{1-S_{11}R}\right).$$

The computational results provided by Theorem 1, summarized in Algorithm 1, justify rigorously the use of this type of approach in the literature, usually implemented by using the Oppenheim and Schafer algorithm exploited in the signal processing field [26] (see Appendix A for more details on this point), for recovering the MM’s electromagnetic parameters [22]. An algorithm derived from the phase-unwrapping procedure of Oppenheim and Schafer that, in principle, can locate and identify the crossings between γ(t) and the principal logarithm branch cut in a more precise way has been developed in [27]. Its pseudo-code is reported in Algorithm 2. A sampled version of $\gamma \left(t\right)≜S_{21}/\left(1-S_{11}R\right)$, [γ(t)] is given as its input. The algorithm checks if [γ(t)] crosses the branch cut of log(·), comparing the position of the points ${\left[\gamma \left(t\right)\right]}_{j-1}$ and ${\left[\gamma \left(t\right)\right]}_{j}$ in the complex plane C−0. The algorithm updates the branch index *p* accordingly, and computes the numerical value of $\left[L\left(\gamma {\left(t\right)}_{j}\right)\right]=log\left({\left[\gamma \left(t\right)\right]}_{j}\right)+i2\pi p$ at point ${\left[\gamma \left(t\right)\right]}_{j}$. If an ambiguity arises, the algorithm stops, the path is re-sampled, using $N=2^{np+1}$ points to this end, and the procedure is performed again.
**Algorithm 1** Phase unwrapping1: a:=0;2: $arg_{\alpha_{0}}\left(\cdot \right):=arg_{\pi }\left(\cdot \right)$;3: $\gamma_{t_{0}}:=\frac{S_{21}\left(a\right)}{1-S_{11}\left(a\right)R\left(a\right)}$;4: ${\left[L\left(\gamma_{t}\right)\right]}_{k=0}=ln\left|\gamma \left(a\right)\right|+i\;arg_{\alpha_{0}}\left(\gamma \left(a\right)\right)$;5: **for** *k*:=1 to n **do**;6:   $t_{k}:=\omega_{k}$;7:   $\gamma_{t_{k}}:=\frac{S_{21}\left(t_{k}\right)}{1-S_{11}\left(t_{k}\right)R\left(t_{k}\right)}$;8:   $p_{k}:=\left(arg_{\alpha_{k-1}}\left(\gamma_{t_{k}}\right)\right)-arg_{\alpha_{k}}\left(\gamma_{t_{k}}\right))/2\pi$;9:   $arg_{\alpha_{k}}\left(\cdot \right):=arg_{\alpha_{k}}\left(\cdot \right)+2\pi p_{k}$;10:   $\left[L\left(\gamma_{t}\right)\right){]}_{k}:=ln|\gamma_{t_{k}})|+i\;arg_{\alpha_{k}}\left(\gamma_{t_{k}}\right))$;11: **end for**

**Algorithm 2** Plane phase unwrapping1: a:=0;2: ${\left[\gamma_{t}\right]}_{0}:=\frac{S_{21}\left(a\right)}{1-S_{11}\left(a\right)R\left(a\right)}$;3: [ut]0:=Re([γt]0);4: [vt]0:=Im([γt]0);5: **for** *k*:=1 to n **do**;6:   $t_{k}:=\omega_{k}$;7:   ${\left[\gamma_{t}\right]}_{k}:=\frac{S_{21}\left(t_{k}\right)}{1-S_{11}\left(t_{k}\right)R\left(t_{k}\right)}$;8:   [ut]k:=Re([γt]k);9:   [vt]k:=Im([γt]k);10: **end for**11: p:=0;12: s_s:=¬ **false**;13: **for** *k*:=1 to n **do**;14:   ${\left[L\left[\gamma_{t}\right]\right]}_{k}:=log\left({\left[\gamma_{t}\right]}_{k-1}\right)+i2\pi p$;15:   **if** ${\left[v_{t}\right]}_{k-1}{\left[v_{t}\right]}_{k}<0$ **then**16:     **if** ${\left[u_{t}\right]}_{k-1}\leq 0\wedge {\left[u_{t}\right]}_{k}\leq 0$ **then**17:       $p=p-\mathrm{sgn}({\left[v_{t}\right]}_{k-1})$18:     **else if** $\left({\left[u_{t}\right]}_{k-1}\leq 0\right.\wedge \left.{\left[u_{t}\right]}_{k}\geq 0\right)\vee \left({\left[u_{t}\right]}_{k-1}\geq 0\right.\wedge \left.{\left[u_{t}\right]}_{k}\leq 0\right)$ **then**19:       s_s:=¬ **true**;20:       **Stop and Re-execute**;21:     **end if**22:   **end if**23: **end for**


### 2.3. Riemann Surfaces and Phase Unwrapping

An alternate and more commonly employed method to overcome the ambiguity afflicting $n_{eff}$ is to resort to the Kramers–Kronig relations [28,29,30]. Because in any physical system the cause cannot precede the effect, the effective permittivities $\epsilon_{eff}$ and $\mu_{eff}$ must obey the causality principle [31,32]. However, the complex refractive index is defined as follows:

(9) $$N_{eff}=\sqrt{\epsilon_{eff}\mu_{eff}},$$

in which at a first glance it could seem that $N_{eff}$ lacks analyticity, because of the presence of branch points in the upper half-plane (UHP) of C due to the zeros of the terms $\epsilon_{eff}$ and $\mu_{eff}$ in this region. Despite this, as demonstrated in [31,32], the term (9) does not have any branch point in the UHP in the case of passive media. This fact allows relating the real and the imaginary parts of $N_{eff}\left(\omega \right)$ via K-K relations and since the term $\kappa_{eff}\left(\omega \right)$ is uniquely determined from relation (4), it allows to determine $n_{eff}\left(\omega \right)$ without ambiguity as follows:

(10) $$\begin{array}{c} n_{eff}\left(\omega \right)-1=\frac{2}{\pi }P\int_{0}^{+\infty }\frac{\omega^{\prime }\kappa_{eff}\left(\omega^{\prime }\right)}{\omega^{\prime 2}-\omega^{2}}d\omega^{\prime }. \end{array}$$

**Figure 1:**
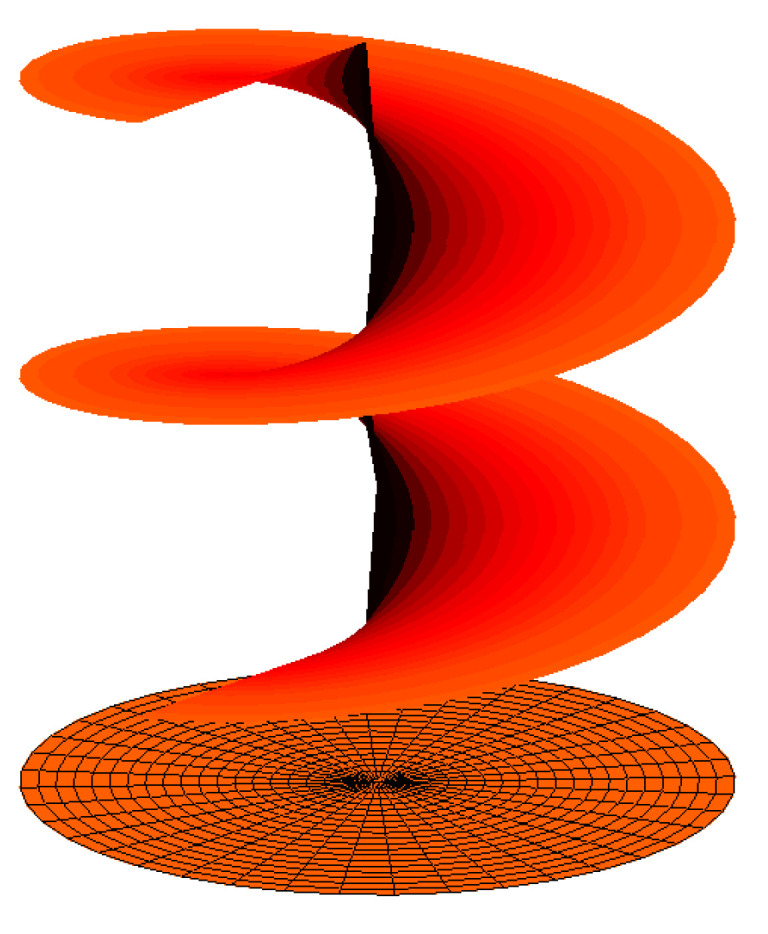
A portion of the Riemann surface S(L) suspended on the unitary disk $D\in \dot{C}$.

To understand how the relation (9) can be exploited in a phase-unwrapping scheme, we have to reconsider the NWR Equation (1) from a more abstract point of view. As discussed in [23], the set of all the analytic logarithms L(·) of (6) makes up a special mathematical object called a global analytic logarithm L. A particular domain of definition is related to L: the Riemann surface S(L) [23]. Its structure is composed of a numerable set of replies of $\dot{C}$, $S_{p}\left(L\right)$, overlayed on top of each other and sorted by the ascending index *p*, suspended above $\dot{C}$, and glued to each other along the slit extending from 0 to −∞, which each sheet owns [23], as shown in Figure 1. If $z^{\prime }$ is a point of $\dot{C}$ and $\overset{˘}{z}$ is the point belonging to $S_{q}\left(L\right)$, for the *q*-th sheet of S(L), which lies above the point $z^{\prime }$ of $\dot{C}$, the value taken by L on $\overset{˘}{z}$, $L_{\overset{˘}{z^{\prime }}}$, is given by [23]:

(11) $$L_{\overset{˘}{z}}=ln\left|z^{\prime }\right|+i\left[arg_{\pi }\left(z^{\prime }\right)+2\pi q\right].$$

Based on the above, if $\overset{˘}{\gamma }$ is the copy on S(L) of the path γ(t) lying in $\dot{C}$, we have from (11) that between the values taken by L(·) at $z^{\prime }\in \gamma \left(t\right)$ and the values assumed by L at $\overset{˘}{z}\in \overset{˘}{\gamma }$, the following relation must hold [23]:

(12) $$L\left(z^{\prime }\right)=ln\left|z^{\prime }\right|+i\left[arg_{\pi }\left(z^{\prime }\right)+2\pi p\right],$$

where, for each $\overset{˘}{z}\in \overset{˘}{\gamma }$, the value assigned to the integer parameter *p* is the index *q* of the Riemann sheet $S_{q}\left(L\right)$ on which $\overset{˘}{z}$ lies. Relation (12) can be used to compute L(·) on $\gamma \left(t\right)≜S_{21}/\left(1-S_{11}R\right)$. Substituting (8) into (12), we have the following:

(13) ImLS211−S11R=argπS211−S11R+2πq.

The unknown indexes *q* that localize the Riemann sheets $S_{q}\left(L\right)$ where the term $S_{21}/\left(1-S_{11}R\right)$ lies can be evaluated considering that from (8) we have the following:

(14) $$n_{eff}k_{0}d=\left[arg_{\pi }\left(\frac{S_{21}}{1-S_{11}R}\right)+2\pi q\right]$$

Inserting (10) into (14), we obtain for the *q* values as follows:

(15) $$q=\frac{1}{2\pi }\left[k_{0}d\left(1-\frac{2}{\pi }P\int_{0}^{+\infty }\frac{\omega^{\prime }\kappa_{eff}\left(\omega^{\prime }\right)}{\omega^{\prime 2}-\omega^{2}}d\omega^{\prime }\right)-arg_{\pi }\left(\frac{S_{21}}{1-S_{11}R}\right)\right].$$

However, from a computational point of view, considering the unavoidable numerical errors that are carried out in the evaluation of the K-K integral (10), the evaluation of the *q* values is better accomplished through the following minimization problem [26]:

(16) $$\begin{array}{cc} \hat{q} & =\underset{q\in Z}{\mathrm{argmin}}\epsilon_{err} \end{array}$$

(17) $$\begin{array}{cc} \epsilon_{err} & =\left|arg_{\pi }\left(\frac{S_{21}}{1-S_{11}R}\right)+2\pi q-\left(1-\frac{2}{\pi }P\int_{0}^{+\infty }\frac{\omega^{\prime }\kappa_{eff}\left(\omega^{\prime }\right)}{\omega^{\prime 2}-\omega^{2}}d\omega^{\prime }\right)k_{0}d\right| \end{array}$$

in which the *q* integers are those minimizing the error, $\epsilon_{err}$, in the absolute value of the difference between the argument of the principal logarithm and the term $n_{eff}k_{0}d$. The two Equations (16) and (17) can be regarded as a numerical integration-like phase-unwrapping approach [26]. As a matter of fact, the above *q* values can be recognized as the integers that allow the phase term $arg_{\pi }\left(S_{21}/1-S_{11}R\right)$ to be continuous, i.e., to unwrap the argument of the principal logarithm, and thus provide the determination of the argument of $L(S_{21}/1-S_{11}R)$, the imaginary part of the right-inverse of which we are searching. Algorithm 3 reports its pseudo-code.
**Algorithm 3** Numerical integration-like phase unwrapping1: a:=0;2: $arg_{\alpha_{0}}\left(\cdot \right):=arg_{\pi }\left(\cdot \right)$;3: $\gamma_{t_{0}}:=\frac{S_{21}\left(a\right)}{1-S_{11}\left(a\right)R\left(a\right)}$;4: ${\left[L\left(\gamma_{t}\right)\right]}_{k=0}=ln\left|\gamma \left(a\right)\right|+i\;arg_{\alpha_{0}}\left(\gamma \left(a\right)\right)$;5: **for** *k*: = 1 to n **do**;6:   $t_{k}:=\omega_{k}$;7:   $\gamma_{t_{k}}:=\frac{S_{21}\left(t_{k}\right)}{1-S_{11}\left(t_{k}\right)R\left(t_{k}\right)}$;8:   $arg_{\alpha }:=\mathrm{Func}\_\mathrm{Kramers}\_\mathrm{Kronig}\_\mathrm{Integral}$9:   $p_{k}:=\mathrm{Func}\_\mathrm{Minimize}\left(arg_{\alpha },arg_{\alpha_{0}}\left(\gamma_{t_{k}}\right)\right)$;10:   $arg_{\alpha_{k}}\left(\cdot \right):=arg_{\alpha_{0}}\left(\cdot \right)+2\pi p_{k}$;11:   $\left[L\left(\gamma_{t}\right)\right){]}_{k}:=ln|\gamma_{t_{k}}\left|+i\;arg_{\alpha_{k}}\left(\gamma_{t_{k}}\right)\right)$;12: **end for**


## 3. Numerical Results

**Figure 2:**
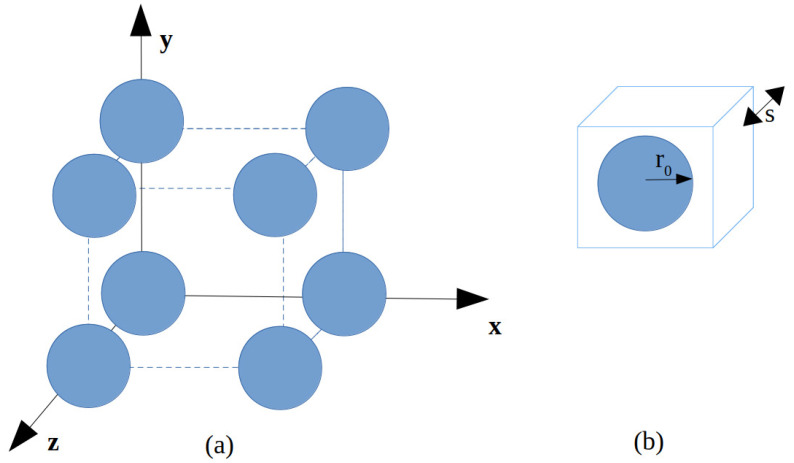
An array of LiTaO_3_ spheres embedded in free space: (**a**) sketch of the considered geometry; (**b**) unit cell containing a single LiTaO_3_ sphere (*s*, cell size; $r_{0}$, sphere radius).

**Figure 3:**
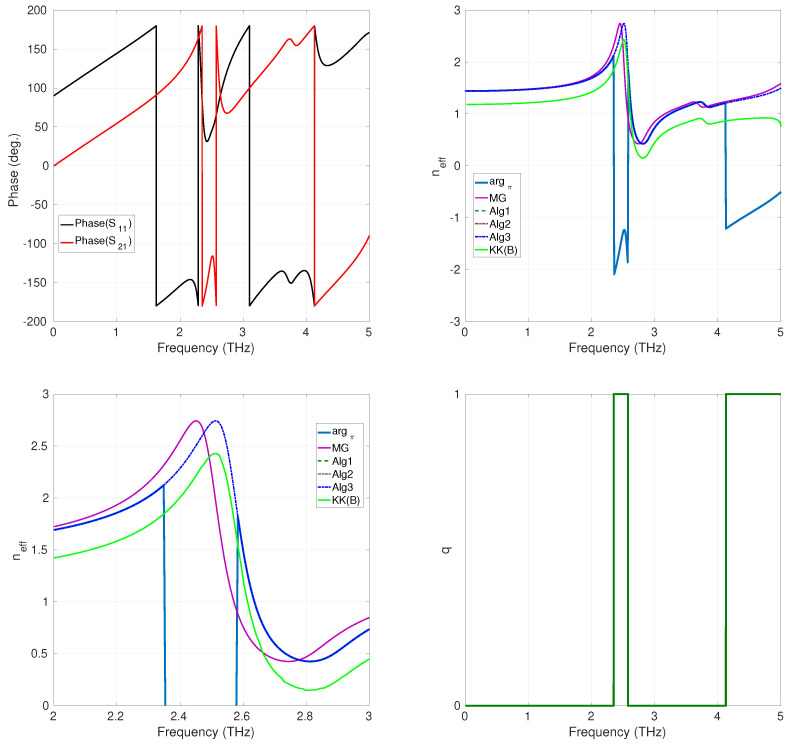
Arrays of LiTaO_3_ spheres, d=30μm; **top-left**: phase of the S-parameters; **top-right**: plot of $n_{eff}$; **bottom-left**: magnification of $n_{eff}$ over the (2,3) THz band; **bottom-right**: Riemann sheets’ *q*-values.

To compare the performance of the phase-unwrapping approaches described in Section 2.2 and Section 2.3 (denoted as Alg1 (phase unwrapping), Alg2 (plane phase unwrapping), and Alg3 (numerical integration-like phase unwrapping), respectively), we have considered the recovering of $n_{eff}$ for a composite realized by a three-dimensional array of lithium tantalate (LiTaO_3_) spheres, considered infinitely large along the x−y axis, and with finite thickness along z, embedded in free space [33]. This is a μ-negative MM for which effective parameters can be analytically checked using the Maxwell–Garnett rules from mixtures [33]. The geometry of the array is reported in Figure 2. Three structures, characterized by a different thickness *d* (which is an integer multiple of the cell size *s*; see Figure 2), were numerically simulated using the software Ansys HFSS, running on an Intel Xeon DP E5405 Quad Core-based workstation. The radius of the LiTaO_3_ spheres was $r_{0}=4\;\mu m$, and the unit cell size was s=10μm [33]. The phase-unwrapping methods were programmed using MATLAB R2023b. The computation of the K-K integral (9) was implemented using the Euler–Maclaurin method described in [34] (the method is denoted as KK(B) in all figures and tables). Figure 3 shows the results for the case d=30μm. The rapid change of the plot of the phase of the S-parameters sampled with $n_{s}=512$ equispaced sampling points in the band (0,5) THz, shown in the top left part of Figure 3, suggests that $n_{eff}$ is affected by the branch ambiguity. This consideration is confirmed by the behavior of $arg_{\pi }\left(\cdot \right)$, reported in the top left part of the same figure, which is clearly discontinuous, and needs to be unwrapped to compute the above parameter. Furthermore, the top right part of Figure 3 shows the comparison between the refraction index $n_{eff}$ computed (i) using the Maxwell–Garnett theory [33], (ii) using the K-K integral, and (iii) using the phase-unwrapping procedures introduced above. We point out that all the considered phase-unwrapping procedures agree with each other and provide results that are close to the $n_{eff}$ obtained by applying the Maxwell–Garnett theory. By contrast, the Kroenig–Kramers integral method reconstructed a refractive index profile that is not comparable with the result provided by the unwrapping methods (see the bottom left part of Figure 3 for details on this point).

**Figure 4:**
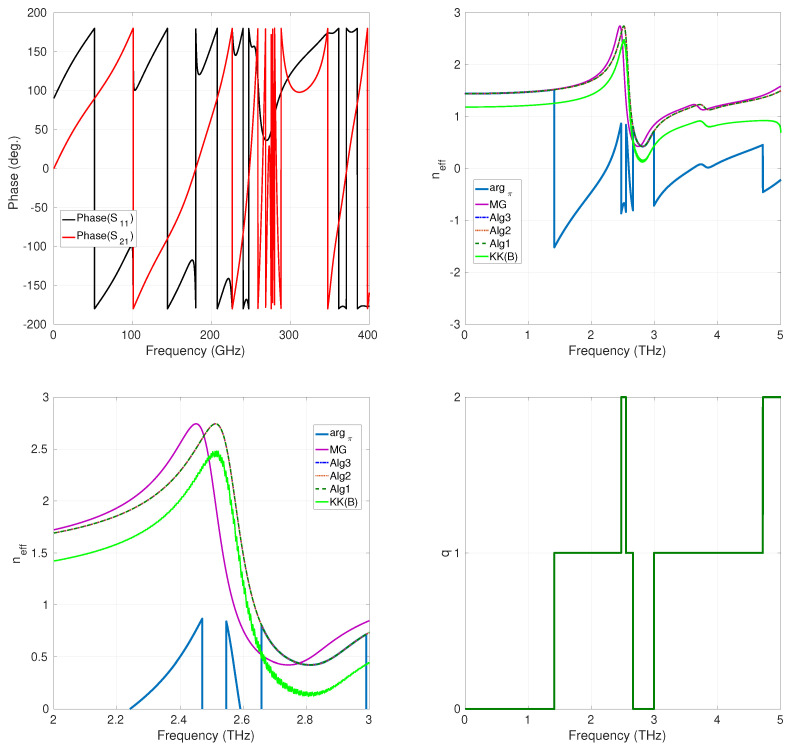
Arrays of LiTaO_3_ spheres, d=70μm; **top-left**: phase of the S-parameters; **top-right**: plot of $n_{eff}$; **bottom-left**: magnification of $n_{eff}$ over the (2,3) THz band; **bottom-right**: Riemann sheets’ *q*-values.

**Figure 5:**
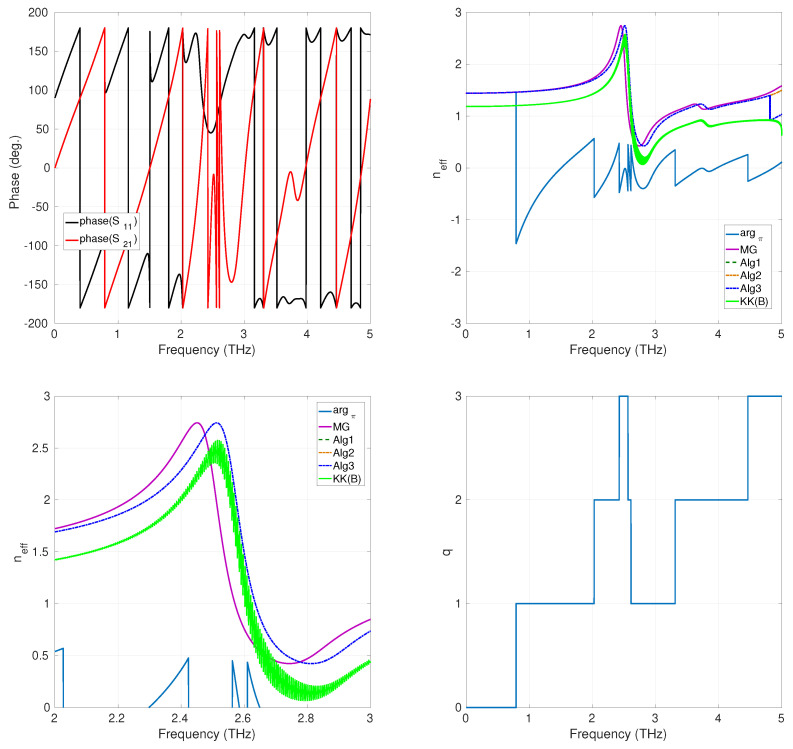
Arraysof LiTaO_3_ spheres, d=130μm; **top-left**: phase of the S-parameters; **top-right**: plot of $n_{eff}$; **bottom-left**: magnification of $n_{eff}$ over the (2,3) THz band; **bottom-right**: Riemann sheets’ *q* values.

In the bottom-right part of Figure 3, the values assumed by the index *q* are reported as a function of the frequency. From these data, we can see that the Riemann sheets involved in the unwrapping process are $S_{0}\left(L\right)$ and $S_{1}\left(L\right)$. Table 1 reports the CPU time needed for each method for the considered case. The results show no particular superiority in terms of this parameter of one method over another. In Figure 4 are reported the results for the case d=70μm. As this structure is thicker than the previous one, we expect the term $S_{21}/1-S_{11}R$ to cross the branching line of the principal logarithm a more significant number of times than in the previous case. This fact is confirmed by the behavior of the phase of the S parameters, in this case sampled with $n_{s}=1024$ equispaced sampling points in the band (0,5) THz. In fact, we can observe that it varies more rapidly than in the previous case. As a consequence, the term $arg_{\pi }\left(\cdot \right)$ results to be more wrapped with respect to the case of d=30μm (see the top-left part of Figure 4. The unwrapping procedures provide results that agree with each other and with those provided for the previously considered less-thick structure (in fact, the refractive index of an assigned media is independent of its thickness). Also, in this case, the result provided by the K-K integral is less accurate (characterized by an oscillatory behavior) than that of the phase-unwrapping approaches provided, as clearly shown in Figure 4, in its bottom-left part, where the magnification of all curves in the (2,3) THz frequency range is reported. $S_{0}\left(L\right)$, $S_{1}\left(L\right)$, and $S_{2}\left(L\right)$ are the Riemann sheets involved. The data reported in Table 2 show that the performances of the CPU time are comparable among methods. Finally, we considered an even thicker structure, with d=130μm. The number of sampling points used in this case was $n_{s}=8046$. The behavior of the argument of the S-parameters and of the argument of the principal logarithm, $arg_{\pi }\left(\cdot \right)$, resulted in a more wrapped result than in the previous cases with d=30μm and d=70μm, as expected (see the top-left and the top-right parts of Figure 5, respectively). For this latter case, the results given by the phase-unwrapping procedures Alg1 and Alg2 are still in good agreement between them and close to the $n_{eff}$ calculated using the Maxwell–Garnett mixing formulas. Regarding the Alg3 procedure, it can be noticed that the provided result is characterized by a discontinuity around f=4.8 THz, a numerical error probably due to the fact that the KK integral is, for the considered case, affected by a large ripple, as shown in the bottom right of Figure 5. Nevertheless, the correction effect offered by the phase-unwrapping algorithm Alg3 on the poor result obtained by calculating the Kramers–Kronig integral is remarkable, as shown in the bottom-left part of Figure 5. $S_{0}\left(L\right)$, $S_{1}\left(L\right)$, $S_{2}\left(L\right)$, and $S_{3}\left(L\right)$ are the Riemann sheets involved in this last case. In terms of CPU time, the Alg3 results are more beneficial compared to the other methods. This result in terms of CPU time can certainly be attributed to the characteristics of this particular method, described in Section 2.2, and is now evident because of the more significant number of $n_{s}$ points used compared to previous cases (see Table 3).

## 4. Conclusions

Based on previous works [22,23], in this paper, after clarifying the connection between the phase unwrapping method and the analytic continuation theory, we have investigated the performances of different phase-unwrapping procedures. To this aim, we have conducted numerical experiments considering a μ-negative MM composite realized with a three-dimensional array of LiTaO_3_ spheres, an artificial composite, intensively considered in the MM literature, for which effective parameters can be analytically checked using the Maxwell–Garnett theory. Numerical results show that the methods based on the simple unwrapping of the phase of the principal logarithm are adequate for all the considered cases, where the unwrapping method based on using the K-K integral can fail if the MM at hand is quite thick. However, this last method has the characteristic of correcting the numerical $n_{eff}$ provided by the K-K integral relation (10), and this is a remarkable result. Regarding CPU time, all unwrapping methods are competitive, although the Alg3 method seems more advantageous for thicker structures. To conclude, we point out that the phase-unwrapping methods, already introduced in the MM literature [19,20,21] but without any theoretical justification, are well grounded in the analytic continuation theory and can be used without them being considered simply empirical techniques for the MM characterization.

## Figures and Tables

**Table 1:** CPU time (s), d=30μm.

$n_{s}$	Alg1	Alg2	Alg3	K-K(B)
512	0.329	0.329	0.349	0.297

**Table 2:** CPU time (s), d=70μm.

$n_{s}$	Alg1	Alg2	Alg3	K-K(B)
1024	0.362	0.343	0.501	0.4981

**Table 3:** CPU time (s), d=130μm.

$n_{s}$	Alg1	Alg2	Alg3	K-K(B)
8192	0.446	0.2936	4.027	3.955

## Data Availability

Data are contained within the article.

## Abbreviations

$\epsilon_{eff}$	Effecive electric permittivity;
$\mu_{eff}$	Effective magnetic permeability;
$N_{eff}$	Complex refractive index;
$n_{eff}$	Effective refractive index;
$k_{0}$	Free space wavenumber;
*d*	MM thickness;
*R*	Reflection coefficient;
*z*	Effective impedance;
$S_{11},S_{21}$	Scattering parameters;
LOG(·)	Complex logarithm;
log(·)	Principal logarithm function;
ln(·)	Natural logarithm function;
|·|	Absolute value function;
$arg_{\pi }\left(\cdot \right)$	Principal argument function;
*p*	Branch index;
$e^{\left(\cdot \right)}$	Complex exponential function;
L(·)	Right inverse function;
L	Global analytic logarithm;
P	Cauchy principal value;
C	Complex plane;
$\dot{C}$	Complex punctured plane;
S(L)	Riemann surface of L.

## Appendix A. Analytic Continuation, Phase Unwrapping, and Homomorphic Systems

In the homomorphic signal processing scope, Oppenheim and Schafer introduced phase unwrapping to make the homomorphic filtering operation possible [35]. Homomorphic filtering is founded on the concept of generalized linear superposition, which is defined as follows:

(A1) $$\phi_{S}\left(c⊙\xi_{i}\left(\tau \right)⊕\xi_{j}\left(\tau \right)\right)=c⊡\phi_{S}\left(\xi_{i}\left(\tau \right)\right)⊞\phi_{S}\left(\xi_{j}\left(\tau \right)\right)$$

where:

(A2) $$\phi_{S}\left(\cdot \right)=\left(\phi_{H^{\prime \prime }}\circ \phi_{H^{\prime }}\right)\left(\cdot \right)$$

are proper input–output functions. The set [⊙,⊕] is made up of an input generalized sum, which takes places between a pair of valid input elements $\xi_{i}\left(\tau \right),\xi_{j}\left(\tau \right)$, and by an input generalized multiplication, which is made between a scalar *c* and an input element $\xi_{i}\left(\tau \right)$. The second set, [⊡,⊞], in a symmetrical way, is composed of an output-generalized sum and an output-generalized multiplication. They operate on scalars *c* and output function elements $\phi_{S}\left(\xi_{i}\left(\tau \right)\right),\phi_{S}\left(\xi_{j}\left(\tau \right)\right)$. In the framework of the homomorphic filtering, the (A1) specializes as follows:

(A3) $$e^{\left(ln\left({\left(\xi_{i}\left(\tau \right)\right)}^{c}\cdot ln\left(\xi_{j}\left(\tau \right)\right)\right)\right)}={\left(\xi_{i}\left(\tau \right)\right)}^{c}\cdot \left(\xi_{j}\left(\tau \right)\right)$$

where $\left[⊕,⊙\right]=\left[⊡,⊞\right]=[\cdot ,{\left(\cdot \right)}^{c}]$ and $\phi_{H^{\prime \prime }}=e^{\left(\cdot \right)};\phi_{H^{\prime }}=ln\left(\cdot \right)$. In this case, the uniqueness and the correctness of the input–output operations are guaranteed by the fact that ln(·) is the inverse of $e^{\left(\cdot \right)}$. To guarantee the same in the case where c∈C and $\xi_{i}\left(\tau \right)$, $\xi_{j}\left(\tau \right)$ are complex-valued functions, Oppenheim and Schafer developed a procedure to restore the continuity of the argument of the principal logarithm $log\left(\cdot \right)=ln|\cdot |+i[arg_{\pi }\left(\cdot \right)]$, avoiding the jumps caused by the crosses with the $R^{-}$ semi-axis, through the computation of the integer parameters *p* needed to compensate these discontinuities [26,35]. Considering the results provided by Theorem 1, we can understand that their procedure is exactly the analytic continuation defined by this theorem, which guarantees the uniqueness of the output operations of a multiplicative homomorphic system by ensuring that the function $\phi_{H^{\prime }}\left(\cdot \right)$ avoids the jumps of $\left[arg_{\pi }\left(\cdot \right)\right]$ caused by crosses with the log(·) branch-cut, which is an admissible right-inverse function l(·) for the complex exponential function $e^{\left(\cdot \right)}$.
